# Microbiome mapping in dairy industry reveals new species and genes for probiotic and bioprotective activities

**DOI:** 10.1038/s41522-024-00541-5

**Published:** 2024-08-02

**Authors:** Francesca De Filippis, Vincenzo Valentino, Min Yap, Raul Cabrera-Rubio, Coral Barcenilla, Niccolò Carlino, José F. Cobo-Díaz, Narciso Martín Quijada, Inés Calvete-Torre, Patricia Ruas-Madiedo, Carlos Sabater, Giuseppina Sequino, Edoardo Pasolli, Martin Wagner, Abelardo Margolles, Nicola Segata, Avelino Álvarez-Ordóñez, Paul D. Cotter, Danilo Ercolini

**Affiliations:** 1 Dept. of Agricultural Sciences, University of Naples Federico II, Portici, NA Italy; 2 Task Force on Microbiome Studies, University of Naples Federico II, Napoli, NA Italy; 3 Teagasc Food Research Centre, Moorepark, Fermoy, Cork, Ireland; 4 Institute of Agrochemistry and Food Technology, National Research Council (IATA-CSIC), Paterna, Spain; 5 Department of Food Hygiene and Technology and Institute of Food Science and Technology, Universidad de León, León, Spain; 6 Department CIBIO, University of Trento, Trento, Italy; 7 Austrian Competence Centre for Feed and Food Quality, Safety and Innovation, FFoQSI GmbH, Tulln an der Donau, Austria; 8 Department for Farm Animals and Veterinary Public Health, Unit of Food Microbiology, Institute of Food Safety, Food Technology and Veterinary Public Health, University of Veterinary Medicine Vienna, Vienna, Austria; 9 Department of Microbiology and Genetics, Institute for Agribiotechnology Research (CIALE), University of Salamanca, Salamanca, Spain; 10 Department of Microbiology and Biochemistry of Dairy Products, Instituto de Productos Lácteos de Asturias - Consejo Superior de Investigaciones Científicas (IPLA-CSIC), Villaviciosa, Spain; 11 Microhealth Group, Instituto de Investigación Sanitaria del Principado de Asturias (ISPA), Oviedo, Spain; 12 APC Microbiome Ireland, University College Cork, Cork, Ireland

**Keywords:** Microbial ecology, Microbiome

## Abstract

The resident microbiome in food industries may impact on food quality and safety. In particular, microbes residing on surfaces in dairy industries may actively participate in cheese fermentation and ripening and contribute to the typical flavor and texture. In this work, we carried out an extensive microbiome mapping in 73 cheese-making industries producing different types of cheeses (fresh, medium and long ripened) and located in 4 European countries. We sequenced and analyzed metagenomes from cheese samples, raw materials and environmental swabs collected from both food contact and non-food contact surfaces, as well as operators’ hands and aprons. Dairy plants were shown to harbor a very complex microbiome, characterized by high prevalence of genes potentially involved in flavor development, probiotic activities, and resistance to gastro-intestinal transit, suggesting that these microbes may potentially be transferred to the human gut microbiome. More than 6100 high-quality Metagenome Assembled Genomes (MAGs) were reconstructed, including MAGs from several Lactic Acid Bacteria species and putative new species. Although microbial pathogens were not prevalent, we found several MAGs harboring genes related to antibiotic resistance, highlighting that dairy industry surfaces represent a potential hotspot for antimicrobial resistance (AR) spreading along the food chain. Finally, we identified facility-specific strains that can represent clear microbial signatures of different cheesemaking facilities, suggesting an interesting potential of microbiome tracking for the traceability of cheese origin.

## Introduction

Cheese production is a powerful method to extend the shelf life of milk and retain its nutritional value^1^. Cheesemaking was probably first performed by chance more than 9000 years ago^2^, but since then a rich variety of cheeses have been developed, differing locally in the type of milk used (raw, pasteurized/thermized, and from different animals), production and ripening technologies and microbial communities naturally selected or deliberately added to achieve the desired sensorial properties.

According to the USDA, > 6 and > 10 million metric tons of cheeses were produced during 2022–2023 in the United States and Europe, respectively (https://fas.usda.gov/data/production/commodity/0240000), and cheese consumption is projected to increase in the next decade^3^, showing that this sector continues to be deep-rooted in human cultural heritage. However, despite the success of the dairy industry, it is estimated that it is the third largest sector in terms of food loss, with ~25% of the dairy production discarded at industrial and household level because of the growth of undesirable microorganisms^4^. Indeed, the ecological properties of cheeses can support the growth of a wide range of microorganisms, including spoilers/pathogens. Notably, *Listeria monocytogenes* can grow well in surface-ripened and fresh cheeses, and several outbreaks involving this microorganism have been reported^5^. However, it is not the sole pathogen associated with cheeses, as others, such as *Staphylococcus aureus* and *Salmonella* spp., can also easily proliferate in the dairy environment^6^.

Cheeses can be spoiled by several microbial species, such as *Pseudomonas putida* and *Ps. fluorescens*. These taxa harbor a broad range of lipolytic and proteolytic enzymes often responsible for off-flavors and the discoloration of cheeses^7–9^. Indeed, most of the spoiling *Pseudomonas* spp. found in dairy products produce the extracellular, heat-stable metalloprotease AprX that targets many different sites in casein^10^. Also, blue pigmentation of fresh and soft cheeses caused by *Ps. fluorescens* has been widely described^11,12^. However, depending on the cheese type, other spoiling bacteria might prevail. For instance, the uncontrolled growth of *Clostridium tyrobutyricum* might lead to late blowing, with the formation of oversized ‘eyes’ (i.e., gas holes) during ripening, and to the development of rancid off-flavors in some Swiss- and Dutch-type cheeses^13^ as well as in hard and long ripened Italian cheeses (e.g., Grana Padano and Parmigiano Reggiano^14^). Furthermore, a strain of *Serratia marcescens* was recently identified as the main responsible of red discolouration in Cabrales cheese^15^. Indeed, although this work is focused only on the bacterial community, also Fungi (yeasts and molds) can be important for the spoilage of cheese^16^, or can actively participate in the manufacturing some types of cheeses.

Although both pathogenic and spoilage microorganisms naturally occurring in milk are easily deactivated by heat treatments commonly performed as preliminary step in most cheese-making processes (e.g., pasteurization or thermization^17^), such microorganisms and their endospores can survive in the processing plant by forming a biofilm, a matrix made of extracellular polymeric substances strongly bound to the surface^18^, which may entrap microbial cells making the cleaning of the processing plants difficult^6^. However, biofilms might also act as a reservoir of pro-technological strains conferring positive sensorial attributes^19^, particularly in cheesemaking. For example, the biofilm on wooden vats is considered as the main source of acidifying Lactic Acid Bacteria (LAB) in the production of the Protected Designation of Origin (PDO) Ragusano cheese^20^ and several other cheeses^21,22^. Also, biofilms on dairy plant surfaces may represent a reservoir of non-starter LAB (NSLAB^23^), potentially contributing to shaping the sensorial profiles of cheeses.

Overall, the residential microbial communities inhabiting the cheese production environment may impact on the quality and safety of the final products, therefore merit an in-depth characterization in order to understand their contribution to shaping the cheese microbiome. To date, a validated procedure to detect and functionally characterize all the microorganisms from the processing environment is lacking, although public investment is currently focusing on this task. Indeed, the recently ended MASTER project (Microbiome applications for Sustainable Food Systems through Technologies and Enterprise, www.master-h2020.eu), granted by the European Union within the Horizon2020 Programme, aimed at developing Standard Operating Procedures (SOPs) for microbiome mapping in food processing environments, to provide food industries with a rapid and effective tool to detect both potentially hazardous and beneficial microorganisms in their facilities. These SOPs will help cheesemakers to quickly detect target microorganisms, as well as to deeply describe the metabolic potential of the microbiome residing in their environment, supporting the overall quality and safety management plans. In the long run, this will lead to a reduction of food spoilage and waste and an improvement of food safety, paving the way to more sustainable food systems.

In the current study, we applied MASTER SOPs to characterize the resident microbiome in 73 facilities, and of 24 cheese types produced at these facilities, from across 4 European countries.

## Results

### Microbiome mapping in dairy industries shows substantially different microbiome compositions from surfaces through raw materials and end products

A total of 1250 samples were collected throughout the study, originating from Austria (*n* = 101), Ireland (*n* = 274), Italy (*n* = 216), and Spain (*n* = 659). Out of all samples, 544 samples were swabs from food contact surfaces (FC, *n* = 308), non-food contact surfaces (NFC, *n* = 199) and operators’s hands/aprons (OP, *n* = 37). Samples of raw milk (*n* = 67), brine (*n* = 44) and whey (*n* = 71) made up the 182 cheese-related material (CRM) samples and the final products (*n* = 524) consisted of samples from the core (*n* = 265) and rind (*n* = 259) of the cheese, which were also sampled before (*n* = 237), during (*n* = 26) and after (*n* = 261) ripening.

**Fig. 1:**
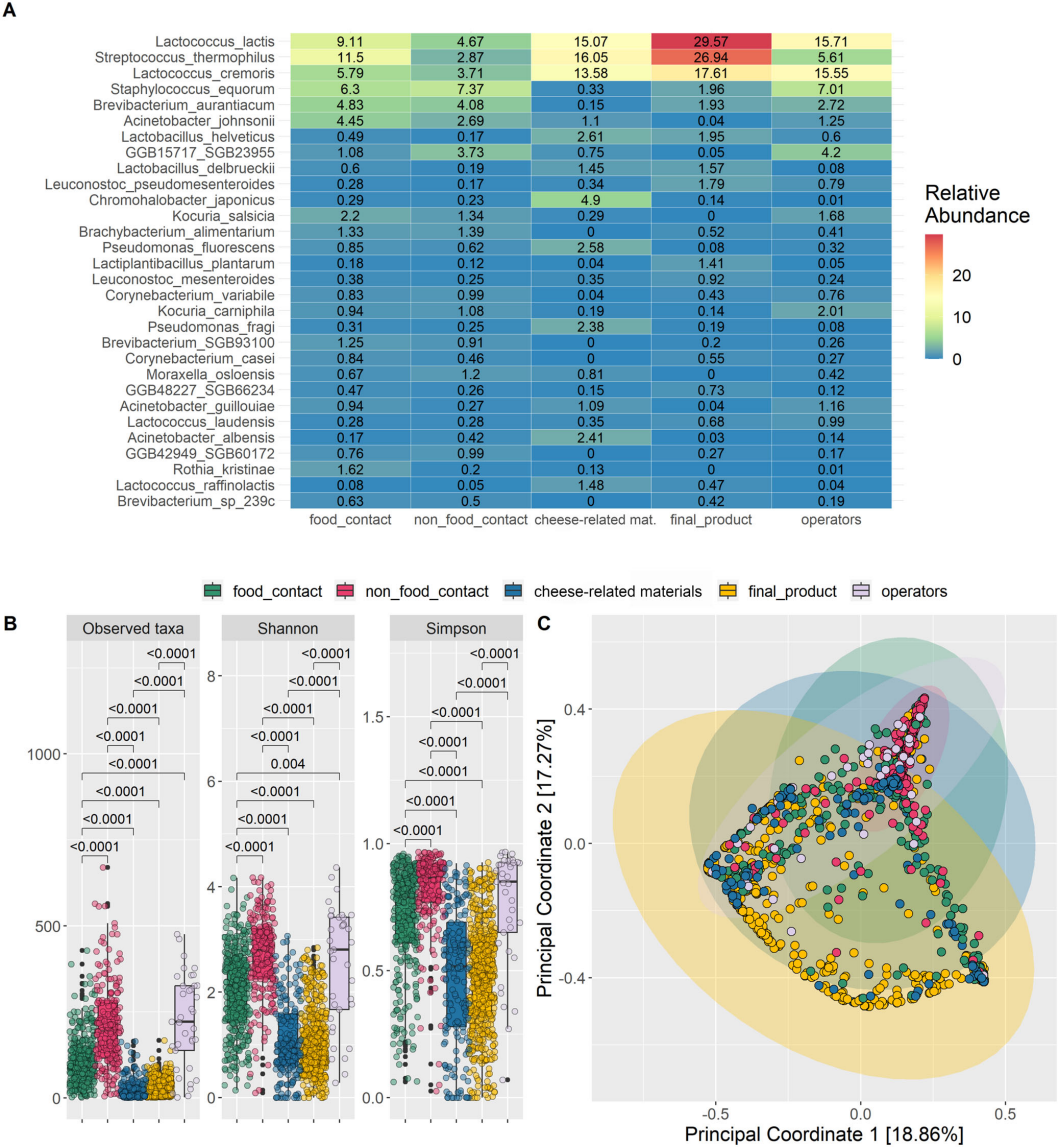
Different taxonomic profiles are present in cheese and environmental swabs. **A**. Taxonomic composition of the 30 most abundant species in all samples, with the average relative abundances of each species in each category. **B**. Alpha diversity, in terms of observed species, Shannon and Simpson analysis of samples by category, and (**C**) beta diversity analysis represented by a Principal-Coordinate Analysis (PCoA) plot of Bray-Curtis distance, with ellipses representing clustering by category. Boxes represent the interquartile range (IQR) between the first and third quartiles, and the line inside represents the median (2nd quartile). Whiskers denote the lowest and the highest values within 1.5 x IQR from the first and third quartiles, respectively. The significance was tested by applying pairwise Wilcoxon test. Average values are obtained from *n* = 308, 199, 37, 524, and 182 biologically independent samples from food contact, non food contact, operators’ swabs, final products and cheese-related materials, respectively. The category “cheese-related materials” groups together milk, brine and whey culture.

From these samples, a total of 239 species were detected at > 0.01% relative abundance. The top 3 species detected, with the highest average relative abundances across all the samples, were *Lactococcus lactis* (18.04% ± 26.8%), *Streptococcus thermophilus* (17.09% ± 28.8%) and *Lc. cremoris* (11.84% ± 22.6%) (Fig. 1A). When stratified into categories, raw materials and final products were dominated by these 3 species (Fig. 1A) and were found to have significantly lower alpha diversity compared to the FC and NFC surfaces and operators’ swabs (*p* < 0.05) (Fig. 1B). In addition, NFC had a significantly higher microbial diversity than FC (*p* < 0.05; Fig. 1B). Apart from the three LAB species, other abundant taxa belonged to *Staphylococcus equorum, Brevibacterium aurantiacum* and *Acinetobacter johnsonii*, that were more abundant on surfaces than in the final products and raw materials (Fig. 1A). Significant differences in the taxonomic composition between all the categories suggested substantially different microbiome compositions across the various types of matrices sampled (PERMANOVA, *p* < 0.05) (Fig. 1C). Considering potentially pathogenic/spoiling taxa, *A. johnsonii* was the most abundant, with average values of 4.5 and 2.7% in FC and NFC, respectively, but at <0.1% in the final products (Fig. 1A). *Staph. aureus* was detected in a total of 92 samples (about 58% of them being final products, while 20% and 10% corresponding to FC and NFC, respectively). However, the overall relative abundance was extremely low, being > 0.1% in only 14 final products, 3 FC and 1 NFC surfaces. Moreover, *Listeria monocytogenes* and *Salmonella* spp. were not detected in any of the samples. *Pseudomonas fluorescens* and *Ps. fragi* were detected among the top 30 species across all categories but were only present in low relative abundances (Fig. 1A, average 0.73% and 0.55%, respectively). These two *Pseudomonas* spp. were generally found in higher relative abundances in the raw materials compared to the other samples, however, in 4 swab samples (3 FC and 1 NFC surface), they were the most abundant species detected (22–45%).

Within the samples representative of the raw materials used for cheese manufacture, a significantly higher alpha diversity was observed in brine, while whey had the lowest diversity (Supplementary Fig. 1). Consistently, whey samples clustered distinctly from samples of raw milk and brine (Supplementary Fig. 1) due to the dominance of 3 main species (*Lc. cremoris*, *Lc. lactis* and *Strep. thermophilus*) that account for an average relative abundance of 82% in all whey samples, and also due to the high abundance of *Chromohalobacter japonicus* (average relative abundance = 20.3%) and *Lc. raffinolactis* (average relative abundance = 3.6%) in brine and raw milk samples, respectively (Supplementary Table 1).

During production, as expected, the microbiome of the cheeses dynamically changed. The number of taxa detected was significantly different between the three time points (before, during and at the end of ripening; Supplementary Fig. 1), with more species observed during ripening, followed by after ripening. The diversity of the cheese microbiome significantly increased during ripening and stabilized towards the end of ripening (Supplementary Fig. 1). However, no clear clustering of the samples was observed (Supplementary Fig. 1). During ripening, different species were found to have significantly higher abundances (Supplementary Table 1), for example, the levels of *Lc. cremoris* and *Lc. lactis* were significantly higher before and after ripening when compared to during ripening, whereas the abundance of *Strep. thermophilus* was significantly higher during, compared to before and after, ripening.

We also compared the microbial community of cheese rind and core, observing a higher diversity on the rind compared to the core (Supplementary Fig. 1). Several species were found at significantly higher relative abundances in the core compared to the rind (Supplementary Table 1). Overall, the core samples had significantly higher abundances of the fermenting LAB *Lactobacillus delbrueckii*, *Lb. helveticus* and *Strep. thermophilus* while significantly higher levels of aerobic bacteria were found in the rind samples (i.e. *B. alimentarium*, *B. aurantiacum*, other unidentified *Brevibacterium* species, *Chromohalobacter japonicus*, *Corynebacterium casei*, *C. variabile*, *Kocuria salsicia* and *Staph. equorum)*. We further compared the cheeses grouped according to the ripening time (<10 days, not ripened; 10–30 days, medium ripened; > 30 days, long ripened) and observed that alpha diversity increased with ripening length (Supplementary Fig. 1). No significant differences were observed in beta diversity. However, cheeses that were not ripened had significantly higher abundances of *Lc. cremoris* and *Strep. thermophilus* compared to the medium- and long-ripened cheeses (Supplementary Table 1**)**.

When grouping cheeses according to the production technology, we found that hard/semi-hard surface-ripened cheeses have the highest alpha diversity and that unripened pasta-filata cheeses were those with the lowest diversity (Supplementary Fig. 1). In addition, pasta-filata cheeses (both unripened and ripened) were dominated by *Strep. thermophilus* at significantly higher relative abundance than other cheeses (Supplementary Table 1). *Lactococcus* spp. were the most abundant taxa in other cheese types: *Lc. lactis* dominated in blue and hard/semi-hard not smear-ripened cheeses, while *Lc. cremoris* in soft cheese and hard/semi-hard, surface-ripened cheeses (Supplementary Table 1**)**.

### The microbiome on dairy industry surfaces has an increased proteolytic activity and the potential to resist GIT passage and colonize the gut

**Fig. 2:**
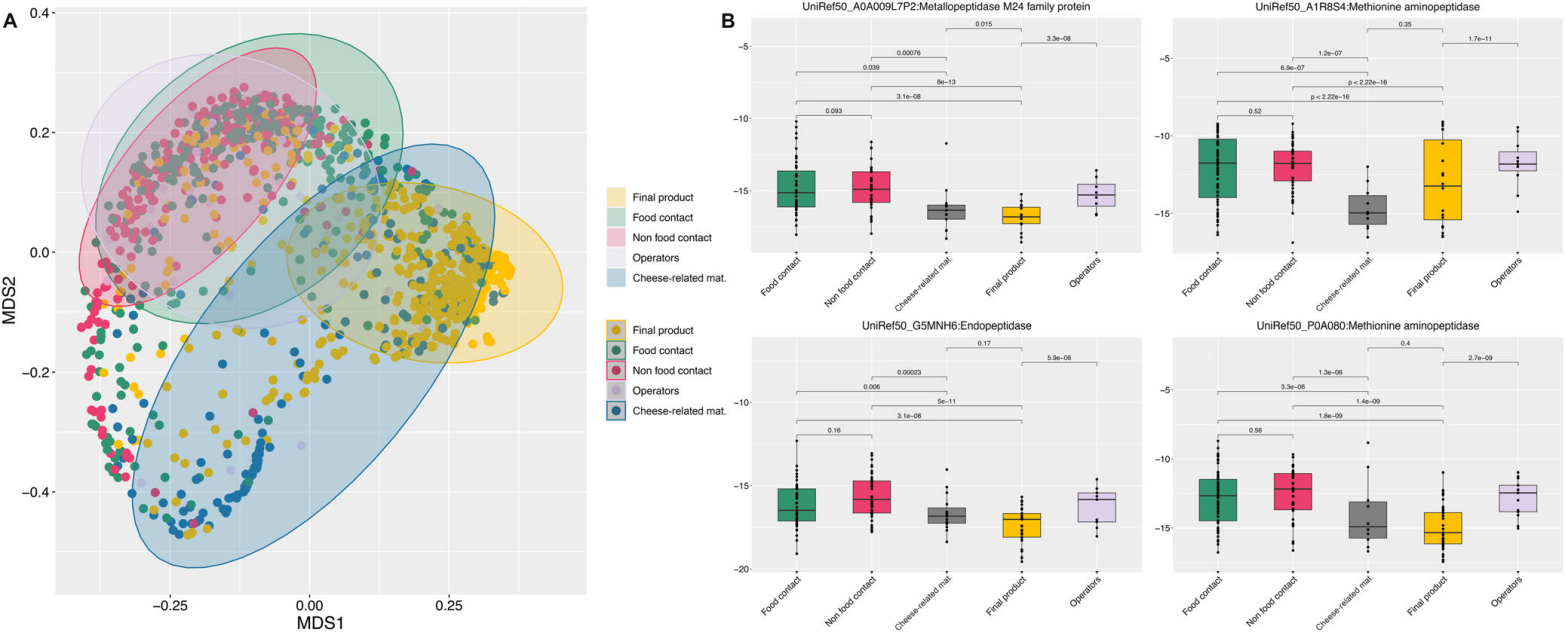
Different functional profiles are present in cheese and environmental swabs. **A** Non-metric Multidimensional Scaling (NMDS) plot based on Jaccard’s distance of functional profiles obtained by HUMAnN. Samples are colored according to the sample type. **B** Boxplots showing the abundance (log values) of UniRef50 genes detected in the different sample groups. Boxes represent the interquartile range (IQR) between the first and third quartiles, and the line inside represents the median (2nd quartile). Whiskers denote the lowest and the highest values within 1.5 × IQR from the first and third quartiles, respectively. The significance was tested by applying pairwise Wilcoxon test. The category “cheese-related materials” groups together milk, brine and whey culture. Average values are obtained from *n* = 308, 199, 37, 524, and 182 biologically independent samples from food contact, non food contact, operators’ swabs, final products and cheese-related materials, respectively.

We investigated functional profiles of microbiomes by mapping short-reads against functional databases through HUMAnN3. The different taxonomic compositions highlighted above (Fig. 1) were reflected in different functional profiles (Fig. 2A), as also highlighted by pairwise comparisons using MANOVA (corrected *p*-value < 0.01 for all the comparisons).

In particular, the microbiome of raw materials and final products showed a higher relative abundance of pathways related to carbohydrate metabolism [e.g., (R,R)-butanediol biosynthesis, Supplementary Fig. 2], while surface-related microbiomes (FC, NFC, operators’ swabs) were characterized by higher abundance of pathways associated with amino acid degradation (e.g., l-leucine, l-arginine and l-ornithine degradation and putrescine biosynthesis), as well as fatty acid oxidation and biotin biosynthesis (Supplementary Fig. 2).

In addition, we further screened gene-level profiles obtained using UniRef50 gene assignment. Consistently, the relative abundance of several genes coding for peptidases (e.g., UniRef50_A0A009L7P2, metallopeptidase family M24; UniRef50_A1R8S4 and UniRef50_P0A080, methionine aminopeptidase; UniRef50_G5MNH6 and UniRef50_G5S167, endopeptidases) were higher in FC and NFC compared with both final products and raw materials (Fig. 2B).

**Fig. 3:**
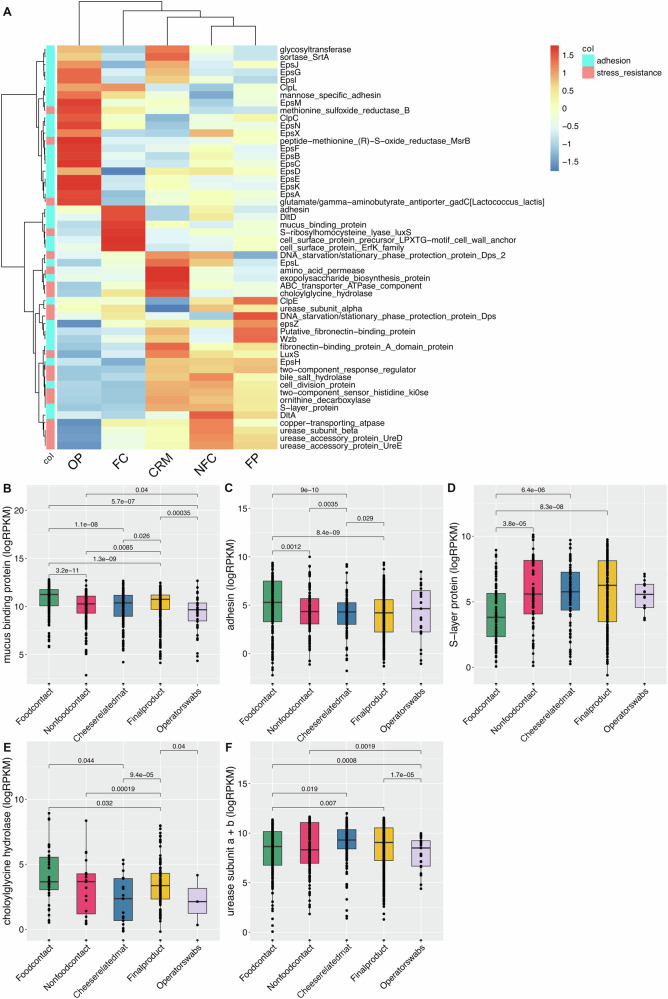
Genes related to gastrointestinal tract transit stress resistance and engraftment encoded in the metagenomes. **A** Heatplot showing the abundance (Reads per Kilobase per Million, RPKM) of predicted genes related to gastrointestinal tract transit stress resistance and engraftment. **B**–**F** Boxplots showing the abundance (log RPKM) in the different sample groups of specific genes. Boxes represent the interquartile range (IQR) between the first and third quartiles, and the line inside represents the median (2nd quartile). Whiskers denote the lowest and the highest values within 1.5 × IQR from the first and third quartiles, respectively. The significance was tested by applying pairwise Wilcoxon test. The category “cheese-related materials” groups together milk, brine and whey culture. Average values are obtained from *n* = 308, 199, 37, 524, and 182 biologically independent samples from food contact, non food contact, operators’ swabs, final products, and cheese-related materials, respectively.

In order to explore the functional potential of the microbiome in greater detail, we predicted microbial genes from assembled metagenomes and mapped them to a custom database including genes related to the resistance to stresses during the gastrointestinal transit, such as the resistance to acids and bile salts, and to the ability of strains to persist in the gut, such as the adhesion to the gut epithelium. Several genes related to stress resistance were enriched in NFC surfaces, final products, and raw materials, while genes related to adhesion prevailed on FC and operators’ swabs (Fig. 3A). Notably, the choloylglycine hydrolase gene, coding for a hydrolase capable of degrading bile salts, urease-encoding genes, needed to counteract acid stress, and some genes involved in the adhesion to the gut epithelium (adhesin, mucus-binding protein) were higher in FC surfaces compared to the final product microbiome, but were also present in NFC surfaces. (Fig. 3B). These results suggest that FC and NFC surface microbiomes are well equipped with traits that may give potential ability to survive during the gastrointestinal transit and persist in the gut, when these microbes are transferred to the food products and ingested.

### The dairy plant microbiome shows a potential protective role through bacteriocin production

**Fig. 4:**
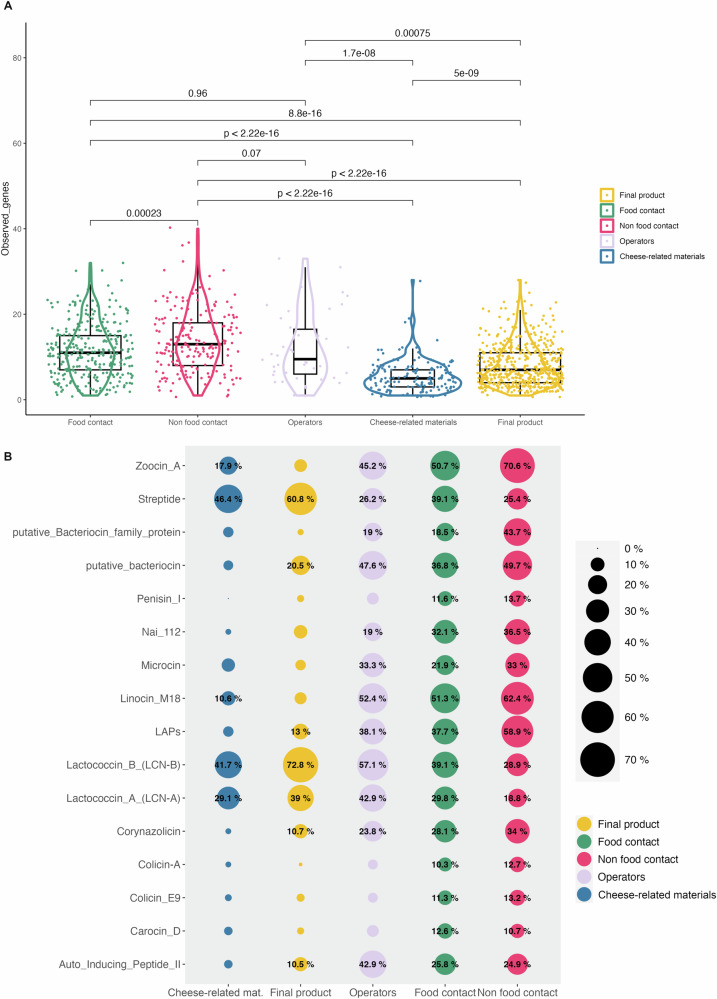
Analysis of the bacteriocins encoded in the metagenomes. The category “cheese-related materials” groups together milk, brine and whey culture. **A** Boxplot reporting the number of observed bacteriocin genes for each sample. **B** Genes observed with a significatively higher frequency in one of the sample groups. Percentages report the proportion of samples from each category testing positive for the selected genes. Boxes represent the interquartile range (IQR) between the first and third quartiles, and the line inside represents the median (2nd quartile). Whiskers denote the lowest and the highest values within 1.5 × IQR from the first and third quartiles, respectively. The significance was tested by applying pairwise Wilcoxon test. Average values are obtained from *n* = 308, 199, 37, 524, and 182 biologically independent samples from food contact, non food contact, operators’ swabs, final products and cheese-related materials, respectively.

To understand if the microbiome within dairy industries hosts microorganisms able to produce bacteriocins, we mapped the contigs against the bacteriocin database distributed with the BAGEL4 tool. A total of 347 genes were detected across all the samples. A clustering between FC/NFC surfaces and ingredients/cheeses can be seen according to the presence-absence profiles of the bacteriocins (Supplementary Fig. 3). We then used the χ2 test on the presence/absence matrix to understand which genes were significantly more prevalent in surfaces or food samples. Excluding the significant genes that were present only in one sample (*n* = 5), 115 genes showed a FDR-corrected *p*-value < 0.05 (Supplementary Table 2). We observed that a significantly higher number of bacteriocin-encoding genes were detected on FC/NFC surfaces compared with ingredients/cheeses (Fig. 4A).

Interestingly, linocin M18-like bacteriocin gene clusters were more prevalent on FC/NFC surfaces (present in 51.3% and 62.4% of the samples, respectively). This pattern was also apparent for corynazolicin- and zoocin A-like clusters. Furthermore, surfaces showed a higher proportion of putative and potentially uncharacterized bacteriocins compared with ingredients and cheeses. On the contrary, lactococcin A-, lactococcin B- and strepide-like clusters were more frequently detected in ingredients and cheeses (Fig. 4B).

### Higher levels of antibiotic-resistance and virulence-associated genes are present on surfaces than cheeses in the dairy plants

**Fig. 5:**
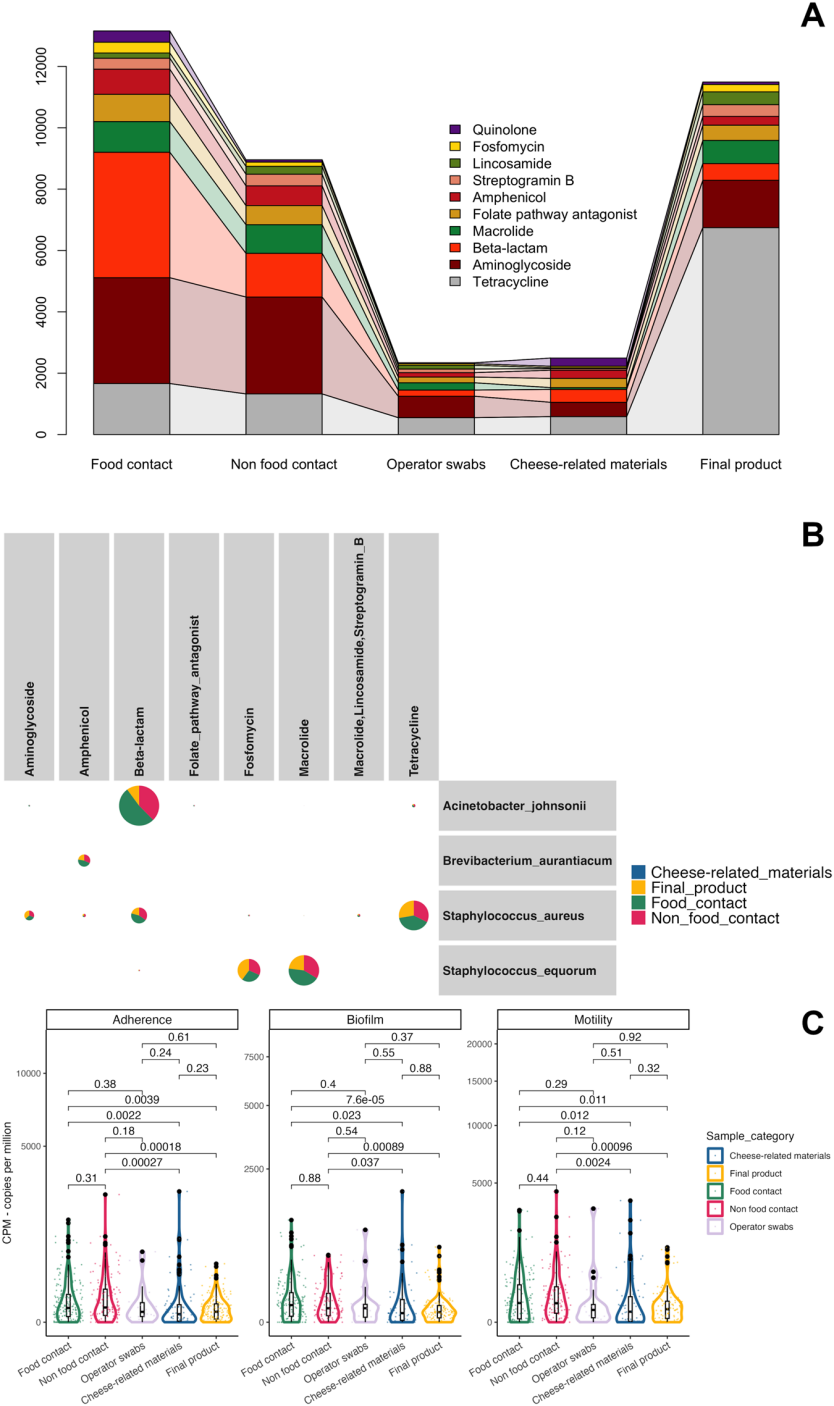
Analysis of antimicrobial-resistance and virulence-associated genes. The category “cheese-related materials” groups together milk, brine and whey culture. **A** Connected barplot showing the variation in the average Copies Per Million (CPM) abundance of each Antimicrobial Resistance Gene (AMRG) family between the classes of samples. **B** Taxonomic assignment of contigs encoding for AMRG. For each AMRG - taxon pair, each slice is proportional to the percentage of contigs reconstructed from a category of samples. The size of each pie is proportional to the number of contigs linked to an AMRG - taxon pair. **C** Sum of the CPM abundance of genes linked with Adherence, Biofilm formation and Motility virulence traits. Statistical differences between the groups were calculated through the Wilcoxon’s rank-sum test. Boxes represent the interquartile range (IQR) between the first and third quartiles, and the line inside represents the median (2nd quartile). Whiskers denote the lowest and the highest values within 1.5 x IQR from the first and third quartiles, respectively. The category “cheese-related materials” groups together milk, brine and whey culture. Average values are obtained from *n* = 308, 199, 37, 524, and 182 biologically independent samples from food contact, non food contact, operators’ swabs, final products and cheese-related materials, respectively.

We detected 32 different AMR genes (AMRG) families in the dairy plant microbiome. Among them, genes associated with resistance to aminoglycosides were the most abundant, with an average CPM of 7.37 ± 25.86, followed by those associated with resistance to tetracyclines (8.59 ± 44.13 CPM) and beta-lactams (5.30 ± 18.44 CPM). A considerable difference was observed between the types of AMRGs associated with surfaces and foods. Indeed, the average CPM abundance of aminoglycoside resistance genes was 2.91 and 2.50 for cheeses and ingredients, respectively, but reached 11.19 and 15.78 in FC and NFC surfaces, respectively (Fig. 5A). The same pattern was observed with respect to other AMRG families, except those encoding tetracycline resistance, which were less abundant on processing plant surfaces than in final product samples (Fig. 5A).

To better understand the distribution of each taxon - AMRG family pair in the cheese production environment, we focused on the taxonomic assignment of the contigs encoding AMRGs (Fig. 5B). Two hundred and thirty-seven out of 292 AMRG-encoding contigs (81.16%) from *A. johnsonii* contained genes associated with beta-lactam resistance. Of these, 48.10 and 34.60% were detected on FC and NFC surfaces, respectively, with the remaining part found in ingredients and products. In addition, 180 and 135 out of 333 AMRG-carrying contigs taxonomically assigned to *Staph. equorum* contained genes associated with resistance to macrolides and fosfomycins, respectively. Of these, the largest proportion of the contigs were detected on FC/NFC surfaces (59.26% for ‘fosfomycin’ resistance genes and 76.11% for ‘macrolide’ resistance genes; Fig. 5B). Then, we computed Spearman’s correlations between microbiome taxonomic profiles and AMRG abundance. Consistently, the relative abundance of *B. aurantiacum*, *Staph. equorum* and *A. johnsonii* were significantly correlated with AMRG families: *Staph. equorum* and *B. aurantiacum* were positively correlated with Fosfomycin and Macrolide AMRG families (Spearman’s rho = 0.68 and 0.67 for *Staph. equorum*, 0.64 and 0.62 for *B. aurantiacum*, respectively; corrected *p*-values < 0.001; Supplementary Fig. 4). Moreover, *A. johnsonii* relative abundance was positively correlated with beta-lactams (Spearman’s rho = 0.60, *p*-value < 0.001). Interestingly, this AMRG family was exclusively linked to this taxon (Supplementary Fig. 4).

We also identified a total of 1056 genes predicted to belong to 13 virulence classes. The ‘effector delivery system’ virulence class was the most frequently detected, with 321 genes, followed by ‘adherence’ (167 genes) and ‘nutritional/metabolic factor’ (151 genes). Interestingly, the top 15 genes, with the highest average CPM abundance, were mostly predicted to be involved in biofilm formation and adherence (Supplementary Fig. 5) and were enriched on FC/NFC surfaces relative to products, suggesting a potential selection for adherence and biofilm formation traits in the cheese production environment. Indeed, the virulence gene showing the highest CPM was PA1464 (also reported as *cheW*), involved in flagellum motility. Consistently, the genes *flgG*, *flgI*, *fliP*, *fliG*, *fleN*, *fliM*, and *flgC*, encoding structural components of flagella or associated regulators, and the genes *pilG* and *pilH*, involved in type IV PilA synthesis, were enriched on FC/NFC surfaces compared with cheeses/ingredients.

The existence of different virulence traits harbored by communities from FC/NFC surfaces and ingredients/products was further confirmed by Non-Metric Multidimensional Scaling (NMDS) (Supplementary Fig. 6; PERMANOVA *p*-value < 0.001). To better understand which virulence classes contributed to the separation of surfaces and foods, we compared the virulence family level CPM values between the sample categories. This analysis highlighted that the ‘Adherence’, ‘Motility’ and ‘Biofilm’ virulence classes were overrepresented on FC/NFC surfaces compared with ingredients/products (Wilcoxon’s rank sum test *p*-value < 0.001; Fig. 5C).

### The dairy plant microbiome includes previously unidentified species

**Fig. 6:**
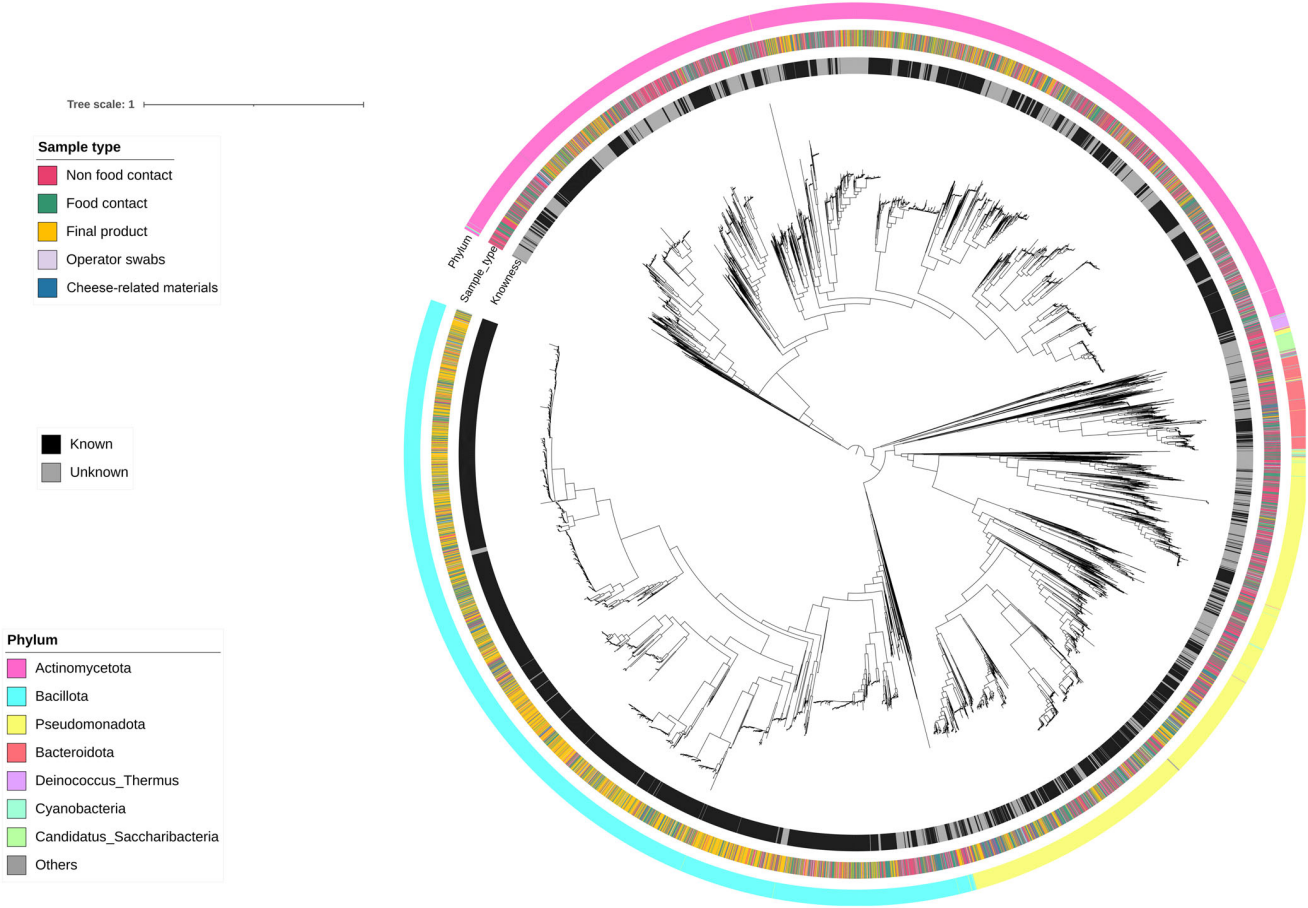
Phylogenetic tree of Metagenome-Assembled Genomes (MAGs) reconstructed from cheeses and dairy environment. Phylogenetic tree of all the MAGs reconstructed in this study, spanning 2111 Species-level Genome Bins (SGBs). From outer to inner, rings are colored according to phylum-level taxonomic assignment, sample type and identification of SGBs at species level (as reported in the Methods). The category “cheese-related materials” groups together milk, brine and whey culture. MAGs have been reconstructed from *n* = 308, 199, 37, 524, and 182 biologically independent samples from food contact, non food contact, operators’ swabs, final products, and cheese-related materials, respectively.

**Fig. 7:**
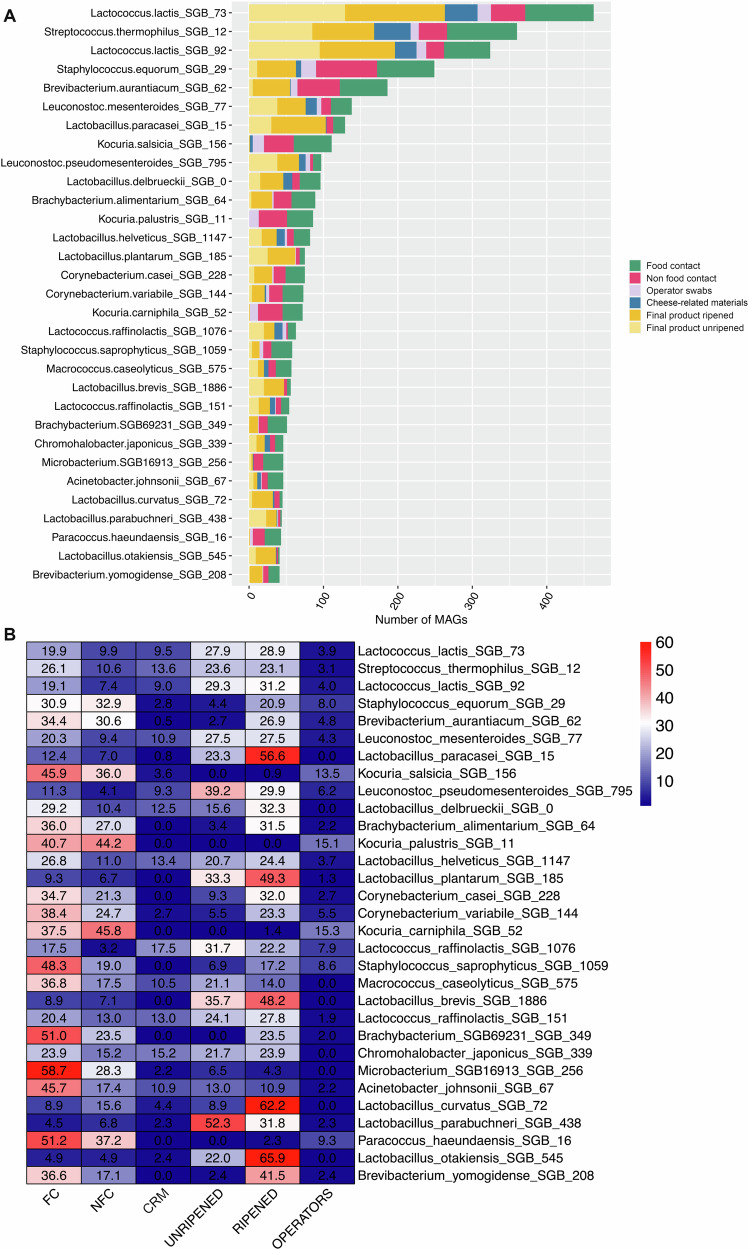
MAGs distribution in cheeses and dairy environment. **A** Bar chart showing the number of Metagenome-Assembled Genomes (MAGs) for the top 30 Species-level Genome Bins (SGBs) reconstructed for each sample group. **B** Heatplot reporting, for each of the top 30 SGBs, the proportion (%) of MAGs reconstructed from each sample group. FC, food-contact surfaces; NFC, non food-contact surfaces; Ripened, ripened cheeses (>30 days); Unripened, unripened cheeses (<30 days); CRM (cheese-related materials), that groups together milk, brine, and whey culture.

We reconstructed a total of 9559 MAGs, which were clustered in 2111 SGBs (Supplementary Table 3). Approximately 63% of the MAGs (6043) corresponded to SGBs with ≥ 10 MAGs (*n* = 171). These prevalent SGBs were considered for further analyses. Among the 171 prevalent SGBs, 130 showed <0.05 MASH genomic distance with one known genome from an isolate or MAG (known SGBs, kSGBs), while 41 were considered as potentially new species (unknown SGBs, uSGBs, with > 0.05 distance from any MAG or isolate genome). Most of the MAGs (49%) were representative of the phylum Firmicutes (recently reclassified as Bacillota), followed by Actinobacteria (Actinomycetota; 39%) and Proteobacteria (Pseudomonadota; 11%). Bacillota MAGs dominated FC surfaces and the unripened cheeses, while Actinobacteriota genomes were mainly reconstructed from raw materials and operator’s swabs (Fig. 6). Interestingly, although 3,028 MAGs were reconstructed from NFC surfaces, only 200 (about 7%) belonged to prevalent SGBs (>10 MAGs), indicating the presence of several low-abundance species on NFC (Fig. 6). Conversely, although a similar number of MAGs was reconstructed from FC (*n* = 3043), about 58% (*n* = 1783) belonged to prevalent SGBs. With respect to uSGBs, 26 (out of 41) were classified at phylum level as being representative of Actinomycetota, including approximately 71% of unidentified MAGs. In contrast, 7 and 6 uSGBs were identified as Pseudomonadota and Bacillota, respectively, while only one was representative of Thermus. SGBs representative of LAB were among the most abundant (Fig. 7A): *Lc. lactis* (two SGBs; SGB_73, *n* = 463 and SGB_92, *n* = 324), *Strep. thermophilus* (SGB_12, *n* = 360), *Leuconostoc mesenteroides* (SGB_77, *n* = 138), *Lacticaseibacillus paracasei* (SGB_15, *n* = 129), *Lactobacillus delbrueckii* (SGB_0, *n* = 96). In addition, several Actinomycetota SGBs were also frequently identified, such as *B. aurantiacum* (SGB_62, *n* = 186), *K. salsicia* and *K. palustris* (SGB_156, *n* = 111 and SGB_11, *n* = 86), *Brachybacterium alimentarium* (SGB_64, *n* = 89), and *C. casei* and *C. variabile* (SGB_228, *n* = 75 and SGB_144, *n* = 74). All these SGBs contained MAGs reconstructed from cheeses and surfaces, both FC and NFC (Fig. 7A). However, unsurprisingly, some differences in the prevalence in the different sample types were observed. *Kocuria* spp. (*K. salsicia*, *K. palustris* and *K. carniphila*) MAGs were typical of environmental samples, mainly being reconstructed from FC and NFC surfaces, as well as from operators’ swabs (Fig. 7B). Conversely, *Staph. equorum* (SGB_29, *n* = 249), *Staph. saprophyticus* (SGB_1059, *n* = 58), *B. aurantiacum*, *C. casei*, *C. variabile* and *B. alimentarium* prevailed in FC, NFC and in ripened cheeses. With respect to the final products, SGBs identified as mesophilic LAB, such as *Lacticaseibacillus paracase*i, *Lactiplantibacillus plantarum* (SGB_185, *n* = 75), *Levilactobacillus brevis* (SGB_1886, *n* = 56), *Latilactobacillus curvatus* (SGB_72, *n* = 46) and *Lentilactobacillus osakiensis* (SGB_545, *n* = 41), were always more prevalent in ripened than unripened cheeses (Fig. 7B).

**Fig. 8:**
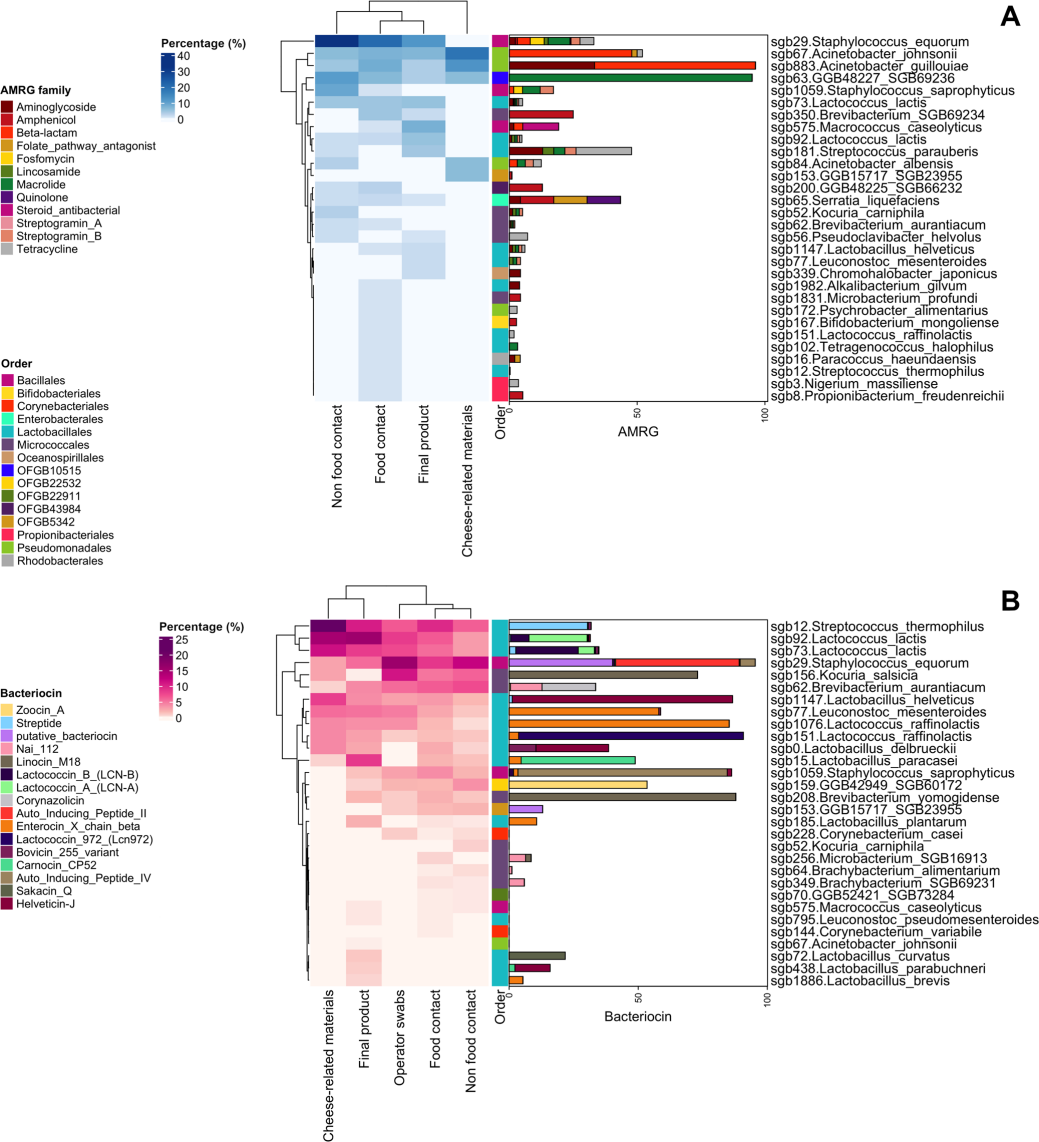
MAGs harbor Antimicrobial Resistance and bacteriocin coding genes. For each group of samples, the heatmaps show the proportion (%) of Metagenome-Assembled Genomes (MAGs) within a Species-level Genome Bin (SGB) containing at least 1 Antimicrobial Resistance (AMR**; A**) or bacteriocin (**B**) coding gene. The side color bar is colored according to order-level taxonomic assignment of the SGB. The bar charts represent the proportion (%) of each AMR or bacteriocin class within the SGB. The category “cheese-related materials” groups together milk, brine, and whey culture.

We further screened MAGs for the presence of AMR or bacteriocin-coding genes (Fig. 8). A total of 554 MAGs contained putative AMRG. Among them, 174 (31.4%) encoded resistance to at least 2 classes of antimicrobial compounds. Most of the potentially antimicrobial resistant MAGs were reconstructed from surfaces (FC, *n* = 224, 40.4%; NFC, *n* = 158, 28.5%), and were identified as *Staph. equorum* (SGB_29, *n* = 57), *A. johnsonii* (SGB_67, *n* = 23) and *A. guillouiae* (SGB_883, *n* = 18). In particular, *Staph. equorum* represented the most abundant potentially antimicrobial resistant SGB on FC, NFC and final cheese samples, accounting for 20.7%, 32.8% and 12.3% of the total resistant MAGs from each group of samples, respectively (Fig. 8A). Although antimicrobial resistant *Staph. equorum* MAGs were most abundant overall, only 57 out of 257 *Staph. equorum* MAGs (22.2%) harbored at least one AMRG. Among these MAGs, genes predicted to encode resistance to macrolides (8.56%), fosfomycin (5.4%), tetracyclines (5.4%) and beta-lactams (5.1%; Fig. 8A) were most common.

Conversely, a high proportion of *A. johnsonii* and *A. guillouiae* MAGs contained putative antimicrobial resistance genes (47.9% and 66.7%, respectively). As expected, almost all of the MAGs from both species contained beta-lactam resistance genes, and 33.3% of the *A. guillouiae* MAGs also contained genes associated with aminoglycoside resistance (Fig. 8A).

3415 MAGs harbored at least 1 gene linked with the production of bacteriocins (as classified in BAGEL4 database): 1098 were reconstructed from FC (32.2%) and 959 from NFC (28.1%), while 1006 and 203 were detected in MAGs from final products and ingredients, respectively. These MAGs mainly belonged to *Strep. thermophilus* (SGB_12, *n* = 234 genomes), the two *Lc. lactis* SGBs (SGB_92, *n* = 234; SGB_73, *n* = 154), *Staph. equorum* (SGB_29, *n* = 193), *B. aurantiacum* (SGB_62, *n* = 125) and *Lacticaseibacillus paracasei* (SGB_15, *n* = 99). More than 50% of the genomes containing bacteriocin-associated genes found in final cheeses belonged to the SGBs reported above. However, the same SGBs accounted for ~36% of the total MAGs harboring potential bacteriocin-producing genes found on surfaces, highlighting that some minor and less prevalent species might contribute to bacteriocin secretion on FC surfaces (Fig. 8B).

Bacteriocin production was a prevalent trait in all the prevalent SGBs. Indeed, 44% and 20% of the *Lacticaseibacillus paracasei* MAGs harbored genes linked with carnocin and enterolysin A biosynthesis, respectively, while 30% of *Strep. thermophilus* (SGB_12) MAGs harbored genes linked with the synthesis of streptide (Fig. 8B).

### Different strains are selected in dairy facilities producing the same cheese type

**Fig. 9:**
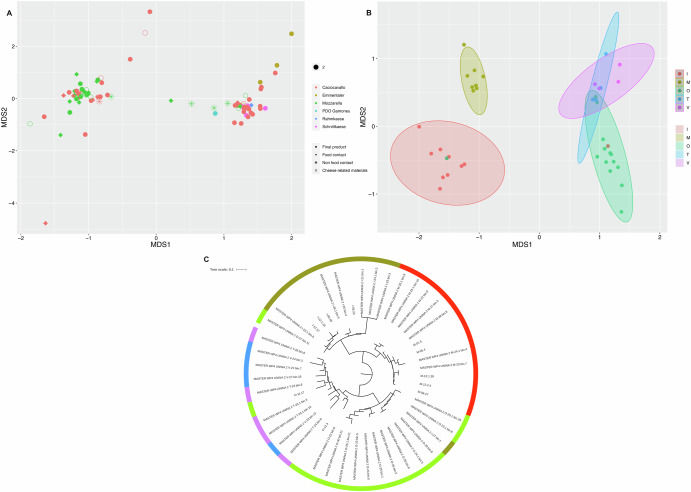
MAGs of *Lb. delbrueckii* can be clustered in different putative strains. Non-metric Multidimensional Scaling (NMDS) based on ANI distance matrix of MAGs belonging to SGB_0 (*Lb. delbrueckii*). **A** All MAGs in SGB_0 are shown. Points are colored according to the cheese type, while different shapes indicate the sample types. **B** Only MAGs reconstructed from Caciocavallo cheese facilities are included and points are colored according to the facility code. **C** Phylogenetic tree of MAGs belonging to SGB_0 reconstructed from Caciocavallo cheese facilities. The category “cheese-related materials” groups together milk, brine and whey culture.

Finally, we looked for genomics variations within the most abundant SGBs. Considering those cheese types for which we sampled several different facilities, we identified in several SGBs the presence of facility-specific strains. For *Lactobacillus delbrueckii* (SGB_0), we identified the presence of two putative strains, but MAGs did not cluster according to the cheese or the sample type (Fig. 9A). However, when considering only MAGs from Caciocavallo dairies (an Italian, ripened pasta-filata cheese), we found that MAGs reconstructed from samples collected in the same factory showed a lower ANI distance and were phylogenetically closer compared with those from different facilities, suggesting the presence of different strains (Fig. 9B-C). This facility-driven clustering of MAGs was observed also for other prevalent SGBs: *Strep. thermophilus* (SGB_12) in Afuega’l Pitu (a Spanish soft-ripened cheese made from acid-coagulated curd) (Supplementary Fig. 7), *Lc. lactis* (SGB_73) in Casín (a Spanish not smear-hard ripened “pasta amasada” – i.e., kneaded curd – cheese) and Caciocavallo (Supplementary Fig. 8) and *Leuc. mesenteroides* (SGB_77) in Casín (Supplementary Fig. 9) dairies.

## Discussion

The surfaces of food industries are inhabited by a complex microbiome, well adapted to the specific processing conditions and microenvironments, that can be considered as residential^24^. Several of these microbes can form biofilms on the surfaces present in facilities, where microbes are embedded in the self-produced extracellular polymeric substances^19,25^. Throughout the food chain, equipment and food contact surfaces provide suitable reservoirs for biofilm formation and accumulation. From these surfaces microbes can be easily transferred to the food product during handling, manufacturing, and storage. In addition, surfaces that are not in direct contact with foods are reservoirs of microbes, which may reach the product. Therefore, the microbiome of the food industry can be regarded as a primary source of microbes for the product. In light of this, mapping its variability across different industries, as well as understanding which microbes, and potential activities encoded within their genomes, prevail in them is of utmost importance in order to improve food quality and safety and reduce food loss in a sustainability perspective.

Several other studies have focused on the microbiome in dairy industries, although the number of companies sampled has always been limited^26–28^. Here we discuss the largest dairy microbiome mapping ever performed, obtained by spanning 73 cheese-making facilities located in 4 different EU countries, producing different types of cheeses and leading to microbiome assessment in 1250 samples. Moreover, the study was performed using a common, standardized and validated protocol for sample collection, processing, sequencing, and data analysis^29^.

We demonstrated that the surfaces of dairy industries harbor a complex microbial community, with > 3000 different species, several of them never identified previously. Consistently with previous reports^27,30,31^, Bacillota, and LAB in particular, can be regarded as part of a core microbiome present in all the dairies and all the different surfaces sampled, with higher relative abundance on food contact surfaces. However, several Actinomycetota (e.g., *Brevibacterium*, *Corynebacterium*, *Kocuria*), Pseudomonadota (e.g. *Acinetobacter*) and non-LAB Bacillota (e.g. *Staph. equorum*) were also identified both in FC and NFC surfaces, as well as in the final products. *Brevibacterium*, *Corynebacterium,* and *Staph. equorum* are well-known inhabitants of the microbiome of surface-ripened cheeses. They actively participate in their ripening^32,33^ and have been previously reported as inhabitants of ripening rooms in traditional cheese production plants^34,35^. *Kocuria* genus includes potential pathogenic species causing bacteremia, previously isolated from cheeses and cheese brine^36,37^, showing the ability to form biofilm^38^ and sometimes reported as carriers of AR genes^38^. In addition, some possible pathogens (e.g., *Staph. aureus*, *A. johnsonii*) were also identified at low abundance, highlighting their ability to persist in the environment and their possible transfer from the surfaces to the cheeses. This also shows the potential of our approach for epidemiological investigations aimed to track the origin of pathogenic strains involved in foodborne outbreaks. However, we also highlighted that pathogens co-exist in the environment with other taxa (e.g., LAB) suggesting the presence of an equilibrium that limits their proliferation. Indeed, a wide range of bacteriocin-producing microbes was identified on cheese industry surfaces (e.g., LAB, *Staph. equorum*, *B. aurantiacum*), harboring genes coding for the biosynthesis of different classes of bacteriocins, including linocin, corynazolicin, zoocin, and lactococcins that were the most common. Genes coding for linocin biosynthesis were particularly abundant on FC and NFC surfaces. Linocin M18 was first identified in *B. linens*, and several in vitro tests highlighted its ability to inhibit the growth of *Listeria monocytogenes*^39^. Notably, this pathogen was not detected in any of the samples analyzed. The production of bacteriocins, besides other mechanisms (e.g., competition for resources, production of other antagonistic substances, development of cheese flavor^40,41^), may exert a protective role, limiting the proliferation of spoiling and pathogenic species within the food industry microbial community. Indeed, the selection of beneficial microbes in food industry surfaces may represent an ecologic and sustainable hurdle against pathogens and spoilers potentially residing in the processing plants, helping in reducing the use of chemicals for disinfection.

Our results also highlighted that the microbiome on dairy surfaces may be a source of technologically relevant activities. Indeed, several genes coding for peptidases, as well as related to aminoacid degradation pathways were enriched on the surfaces, suggesting that surfaces can be an important source of microbes and metabolic activities that are important for cheese ripening and flavor production, as often reported^42^.

We also showed that the resident microbiome in dairy plants harbors genes related to the ability to potentially survive the stress conditions encountered during the gastrointestinal tract transit (e.g., acids, bile salts) and to engraft in the gut, becoming part of the gut microbiome. Previous reports suggested that LAB strains from fermented foods show similarity with those present in the human gut^43^ and that they have the genomic repertoire that allow them to persist in the gut^44^. Our study firstly highlighted that this can be translated also to the dairy plant microbiome, that, once transferred to the products, may represent a source of microbes for the human gut. This can be particularly of concern since we also found that the microbiome in dairy surfaces also harbors a wide pattern of AMR genes, where tetracycline, beta-lactam, and amphenicol resistance genes are the most abundant. Interestingly, these are the most common classes of AMR genes found in pathogenic strains isolated from milk and in milk microbiome^45–47^, as well as the residual antibiotics commonly found in retail milk from different countries^48^. Recently, fermented foods, particularly cheeses, were highlighted as a hotspot for AMR transfer^49^. Our study confirms this evidence and highlights that the food processing environment may be involved in this process. Indeed, our results suggest that the FC and NFC surfaces in dairies have a complex resistome that may reach the final product, and, upon ingestion, AMR genes may be transferred to the gut microbiome or to pathogens through horizontal gene transfer events.

Finally, although most of the dairy plants we sampled produce PDO cheeses and therefore apply the same standardized production process, we demonstrated that a strain-level selection may occur in different facilities producing the same cheese (e.g., the Italian PDO Caciocavallo Silano; the Spanish PDO “Afuega’l Pitu” and PDO “Casín”). Different strains may come from raw materials (e.g., milk) or starter cultures used during manufacturing (commercial or natural starters), but their presence in environmental swabs suggests that these strains are able to persist in the dairy plant and take over during the manufacture and ripening process. Strain diversity in cheeses may lead to different organoleptic attributes^50,51^. Therefore, these results support the idea that cheeses from different producers may have peculiar traits. Moreover, mapping strain diversity across PDO cheese producing plants may be useful to design novel strategies for origin tracking based on microbiome fingerprinting. However, we have to point out some limitations of our study that might have partially influenced the results. Firstly, microbial biomass was not quantified, so the comparison of results between samples with high (e.g., cheeses) and low (e.g., surfaces) biomass might be biased. In addition, our study is based on DNA analysis. Therefore, we cannot exclude that part of this DNA might come from dead or damaged cells.

We provided a comprehensive mapping of the microbiome across dairy plants in different EU countries. Our study demonstrated that the cheese industry harbors a highly complex microbiome, selected by the specific technological process and that can be transferred to the product. We highlighted that it may exert a positive role, actively participating in cheese flavor production and inhibiting pathogen development. However, the results also suggested that dairy industry surfaces can be an important reservoir of AR strains that may contaminate the product and survive during the gastrointestinal passage, reaching the gut microbiome.

The integration of procedures for microbiome mapping in the dairy industry can support the overall quality management strategy. This may help to improve cheese quality and safety, without compromising the specificity of each product, the link with traditional practices and with the geographical area of production.

## Methods

### Sampling, DNA extraction, and metagenome sequencing

Seventy-three facilities producing different types of cheeses and located in 4 EU countries (Austria, *n* = 6; Ireland, *n* = 15; Italy, *n* = 16; Spain, *n* = 36) were visited (February-October 2020) after the completion of routine cleaning procedures (Supplementary Table 4). Surfaces, cheese-related materials (milk, whey culture, brine) and final products (fresh, medium and long-ripened cheeses) were collected according to the MASTER SOP-01 (https://www.master-h2020.eu/SOPs.ht ml, ref. ^29^). For all cheeses except Buffalo Mozzarella, samples of cheese rind and core were analyzed separately. Food contact (FC) and non-food contact (NFC) surfaces from the facilities were sampled using Whirl-Pak Hydrated PolyProbe swabs (Whirl-Pak, Madison, Wisconsin, US), covering an area of about 1 m^2^, or a sampling unit (e.g., one table, one sink). FC included (according to the type of cheese and the industry set up) draining tables, curd vats, curd shredders, molding machines and ripening shelves, while NFC included walls, floors, sinks and drains. When available, different areas in the facility were sampled: processing, ripening, and packing rooms. In addition, swabs were collected from the hands/aprons of the employees working on the production line. Five swabs from each sampling point were collected. A list of the samples collected in each country is provided as Supplementary Table 5. In the laboratory, the 5 swabs from each surface were pooled together and 10 mL of Phosphate Buffered Saline (PBS) 1X were added to each pool. After homogenization in a stomacher, the cell suspension was collected and centrifuged at 5000 x g for 15 minutes. DNA extraction was performed from the cell pellets using the PowerSoil Pro Kit, adopting a previously validated modified version of the standard protocol optimized for food processing environments and low biomass samples^29^. Metagenomic libraries were prepared using the Nextera XT Index Kit v2 (Illumina, San Diego, California, United States), and metagenome sequencing was performed on an Illumina NovaSeq platform, leading to 2 × 150 bp reads. Negative controls were collected both at the industry and in the laboratory, leaving a pool of 5 swabs exposed to the air for 1 minute. Negative controls were then processed as reported for the other samples. However, DNA yield was really low (< 1 ng/μl), and library preparation failed for all the negative controls.

### Reads pre-processing, taxonomic, and functional profiling

The raw reads were filtered using a validated pipeline (available at https://github.com/SegataLab/preprocessing) composed of three sequential steps: i) discarding of low-quality (quality<20), short (L < 75 bp) and with-ambiguous-nucleotides (n < =2) reads using Trim Galore v0.6.6 (https://www.bioinformatics.babraham.ac.uk/projects/trim_galore/); ii) identification and removal of human (HG19 human genome release) and bacteriophage phiX174 DNA (Illumina spike-in) contamination by mapping reads against target reference genomes through BowTie2 v2.2.9^52^ (with parameter –sensitive-local) and iii) splitting of remaining reads into forward, reverse and unpaired files).

Taxonomic and functional profiles were obtained using short reads with MetaPhlAn^53^ (version 4.0.2 (qstat = 0.2) with the marker database ChocoPhlAn v202204) and HUMAnN^54^ (version 3.0, options --metaphlan-options “-t rel_ab --index v30_CHOCOPhlAn_201901”), respectively.

### Assembly, Metagenome-Assembled Genomes (MAGs) reconstruction and analysis

High-quality reads were assembled independently using MEGAHIT^55^ v. 1.2.1 (with default options) and contigs > 1000 bp were used to predict genes by using MetaGeneMark^56^ v. 3.26. Predicted genes were aligned (using DIAMOND^57^ v. 2.0.4 and the option –very-sensitive), against a custom database including known microbial genes potentially involved in stress resistance and adhesion to the gut epithelium^58^. Accession numbers and gene identification are reported in Supplementary Table 6. An e-value cutoff of 1e^−5^ was applied, and a hit was required to display > 95% of identity over at least 50% of the query length.

Metagenome assemblies were processed through TORMES^59^ v. 1.3.0 (options --gene_min_id 80 --gene_min_cov 80) to detect antimicrobial resistance (AMR) and virulence genes. For AMR identification, TORMES relies on three databases, i.e., ResFinder^60^, CARD^61^ and ARG-ANNOT^62^, whereas virulence genes were detected through the VFDB^63^. Only hits showing >80% identity over >80% of the query length were retained.

To estimate the AMR/virulence genes abundance, raw reads were filtered against the ResFinder database through BowTie2^52^ v2.2.9 using the parameters reported above. The resulting sam files were filtered through an in-house script (https://github.com/SegataLab/MASTER-WP5-pipelines/blob/master/07-AMR_virulence_genes/count_reads.rb), then Copies Per Million (CPM) were computed by normalizing the n. of mapped bacterial reads (as estimated by viromeQC^64^) for the total number of reads in the metagenome. In addition, BAGEL4^65^ was used to identify the genes predicted to encode bacteriocins in the metagenomes.

To obtain the gene abundance, short reads were mapped to the genes using BowTie2 (options: –very-sensitive-local –no-unal) and the number of mapped reads was normalized using the RPKM method (reads per kilobase per million mapped reads), considering the formula (number of hits for each gene/gene length)/total number of mapped reads per sample^66^.

Contigs shorter than 1,000 bp were discarded. The coverage for the remaining contigs was calculated by aligning them against the original reads with BowTie2 v2.2.9 and used for binning through MetaBAT 2 ^67^ v. 2.12.1. The quality of Metagenome-Assembled Genomes (MAGs) was estimated with CheckM^68^ v. 1.1.3: only MAGs with > 50% completeness and < 5% contamination were retained for further analyses. MAGs included in this study were included in a > 1 M genomes database, MetaRefSGB vMar22, where they were clustered according to genetic distances. These MAGs were part of a wider catalog recently developed within the MASTER project. Pairwise genetic distances between all genomes were calculated using MASH^69^ (version 2.0, option “-s 10,000” for sketching). A MASH distance < 5% from any of the database genomes was considered to place the MAG within the relative species-level genome bin (SGB). When a MAG showed > 5% distance from any of the reference genomes, it was considered a novel species (unknown SGB, uSGB). In this case, the taxonomic assignment was made at genus (>5 and <15% distance), family (> 15 and < 25% distance), or phylum (> 25% distance) level. Thresholds previously reported were used^70^. To define subspecies within each SGB, pairwise average nucleotide identities (ANI) distances between MAGs were calculated through FastANI^71^ (using default options), while phylogenetic analyses were performed using PhyloPhlAn^72^ (options --diversity low and --accurate; version 3.0.67). Phylogenetic trees were visualized using iTol^73^.

### Statistical analyses

All the statistical analyses were performed in a R environment (https://www.R-project.org/; version 4.2.2). The relative abundances of taxonomic/functional profiles and the genes’ CPM/RPKM were compared through the Wilcox-Mann-Whitney test using the R function ‘wilcox.test’. Jaccard and Bray-Curtis distance matrices were computed with the function ‘vegdist’ (‘vegan’ package), then Principal Coordinates Analyses and Nonmetric Multidimensional Scaling (NDMS) were performed through ‘cmdscale’ and ‘metaMDS’, respectively (both from the ‘base’ R package). Permutational MANOVA (function ‘adonis2’ from the ‘vegan’ R package) highlighted if the groupings of samples according to the distance matrices were statistically significant. Pairwise comparisons between permutational MANOVA results were done with the function ‘pairwise.perm.manova’ from the ‘RVAideMemoire’ package. The χ2 test was applied on contingency tables built from presence-absence matrices to detect genetic features significantly more prevalent in specific groups of samples. All the *p*-values were FDR-adjusted when needed. Also, Spearman’s ρ between AMR-taxa and AMR-virulence abundances were computed with the function ‘correlate’ from the ‘corrr’ R package and plotted using ‘cor.graph’ (for AMR-taxa correlations) and ‘corrplot’ (for AMR-virulence correlations). Bar/pie charts and box/scatter/violin/correlation plots were generated using the ‘ggplot2’ and ‘ggpubr’ R packages, whereas heatmaps were plotted through ‘pheatmap’ (using the euclidean distance and the complete-linkage clustering method).

### Reporting summary

Further information on research design is available in the Nature Research Reporting Summary linked to this article.

### Supplementary information

Supplementary Figures

Supplementary Table 1

Supplementary Table 2

Supplementary Table 3

Supplementary Table 4

Supplementary Table 5

Supplementary Table 6

Reporting summary

## Data Availability

The raw sequence reads generated from samples collected in Italy, Spain and Austria have been deposited in the Sequence Read Archive (SRA) of the NCBI under accession number PRJNA997821, whereas those from Irish facilities are available in the European Nucleotide Archive database under accession number PRJEB63604. All software used are freely available for download.
