# Supplementary material for: Microbiome mapping in dairy industry reveals new species and genes for probiotic and bioprotective activities
Source: NPJ Biofilms Microbiomes. 2024 Aug 2;10:67. doi: 10.1038/s41522-024-00541-5 (PMC11297241; doi:10.1038/s41522-024-00541-5)
Supplement: Supplementary file 1 — Supplementary Figures [file 41522_2024_541_MOESM1_ESM.pdf]

Supplementary Figures

Microbiome mapping in dairy industry reveals new species and genes for probiotic and bioprotective activities

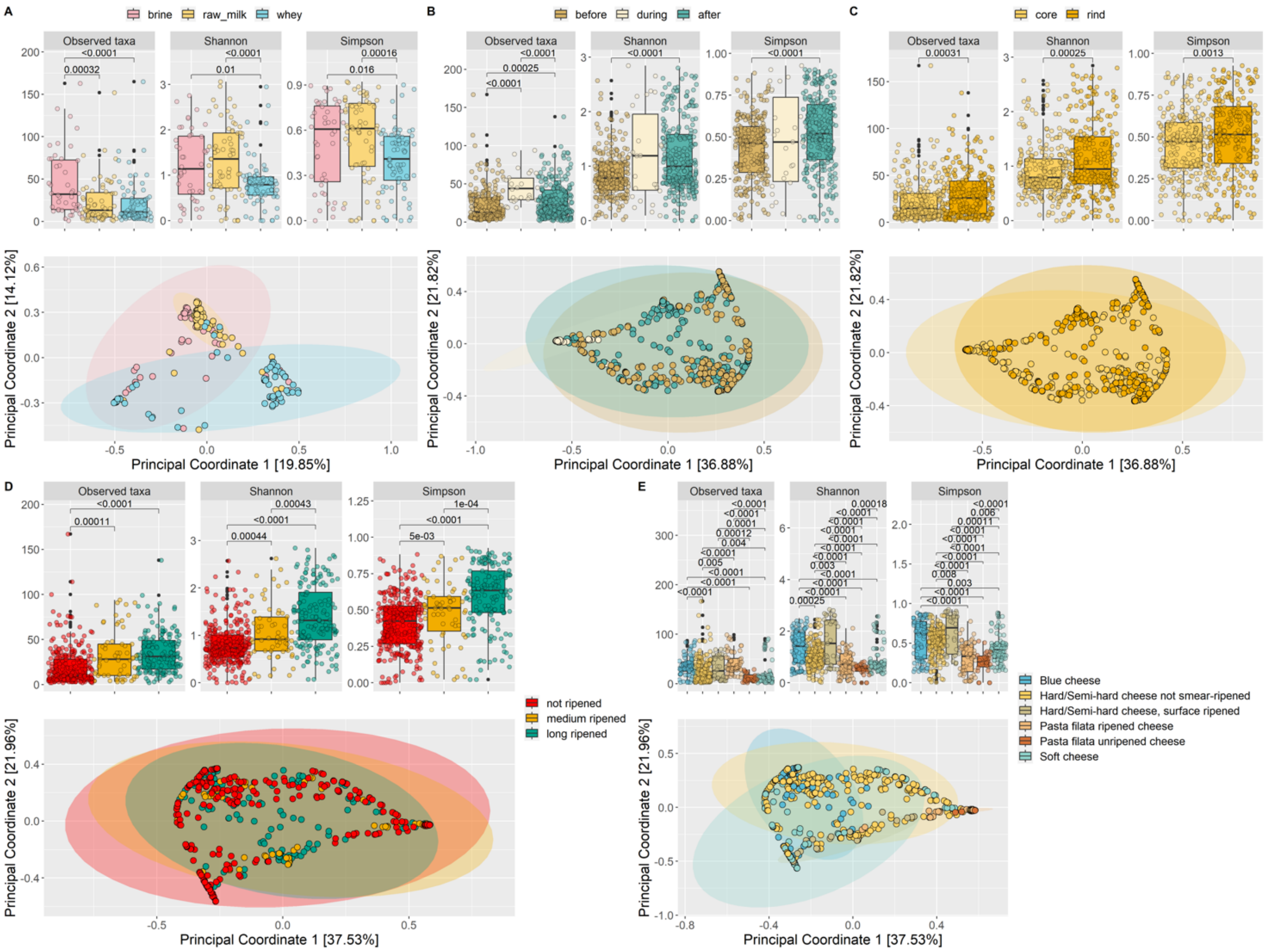

Supplementary Figure 1. Alpha and beta diversity of species level bacterial profiles of (A) raw materials, including brine, raw milk and whey, (B) cheese at different ripening stages, before, during and after, (C) the core and rind of final cheeses, (D) the cheeses grouped according to ripening time and (E) between the different production technologies. Alpha diversity analysis includes observed species, Shannon and Simpson metrics, and beta diversity Bray-Curtis PCoA plots with ellipses indicating clustering of different groups.

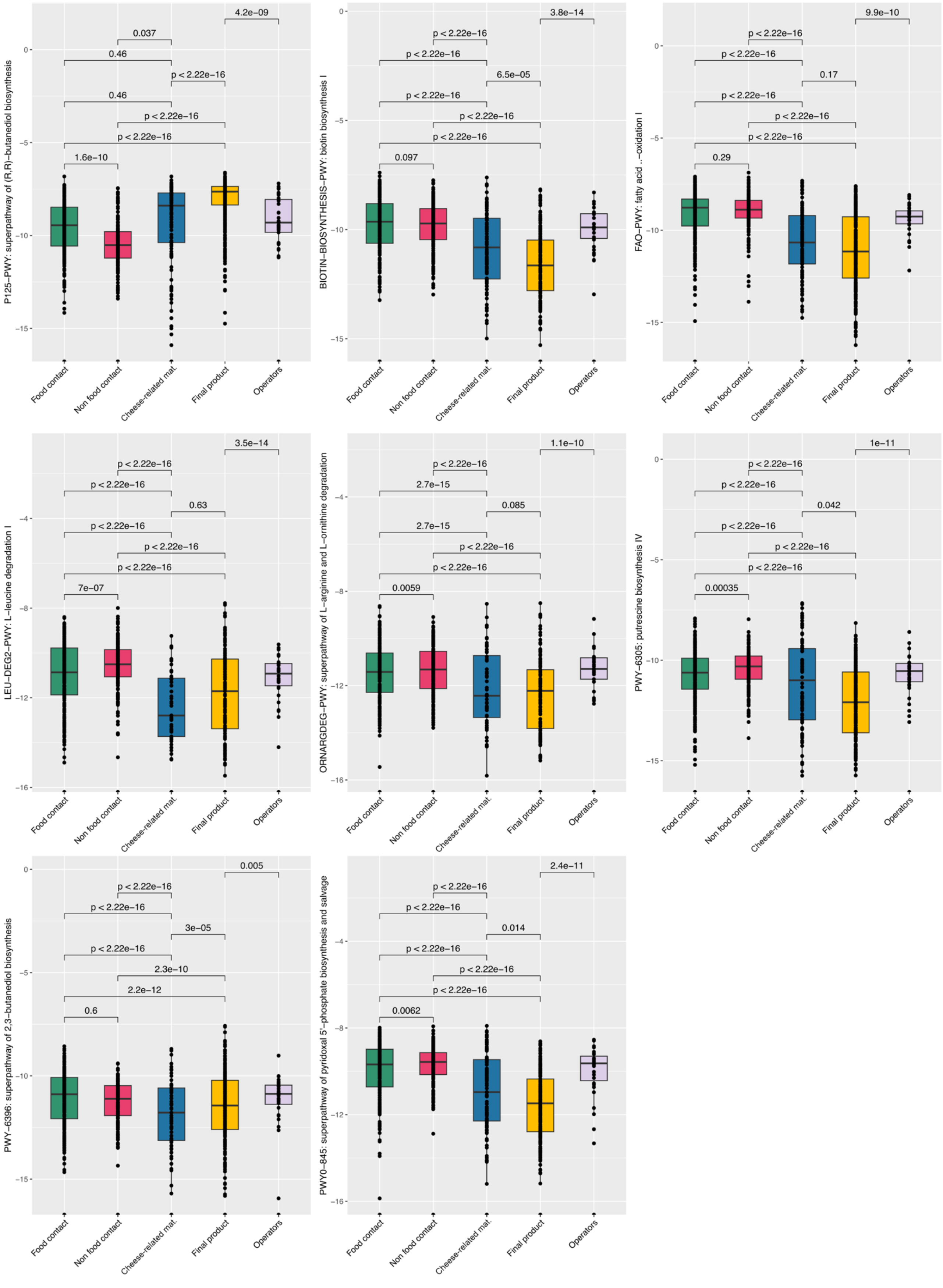

Supplementary Figure 2. Boxplots showing the abundance (log values) of metabolic pathways detected in the different sample groups. The category “cheese-related mat.” groups together milk, brine and whey culture.

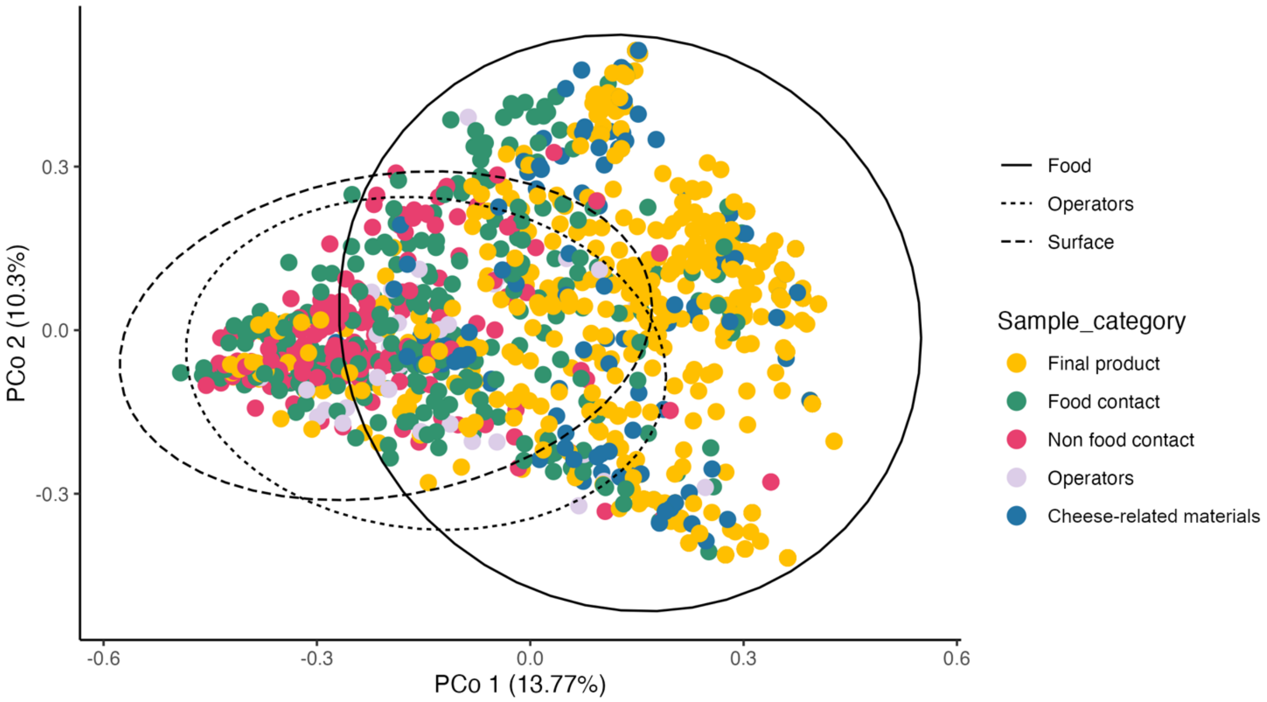

Supplementary Figure 3. Principal Coordinate Analysis performed on the presence-absence profiles of bacteriocin-producing genes. The Jaccard index was used to compute dissimilarities between each pair of samples. The category “cheese-related materials” groups together milk, brine and whey culture.

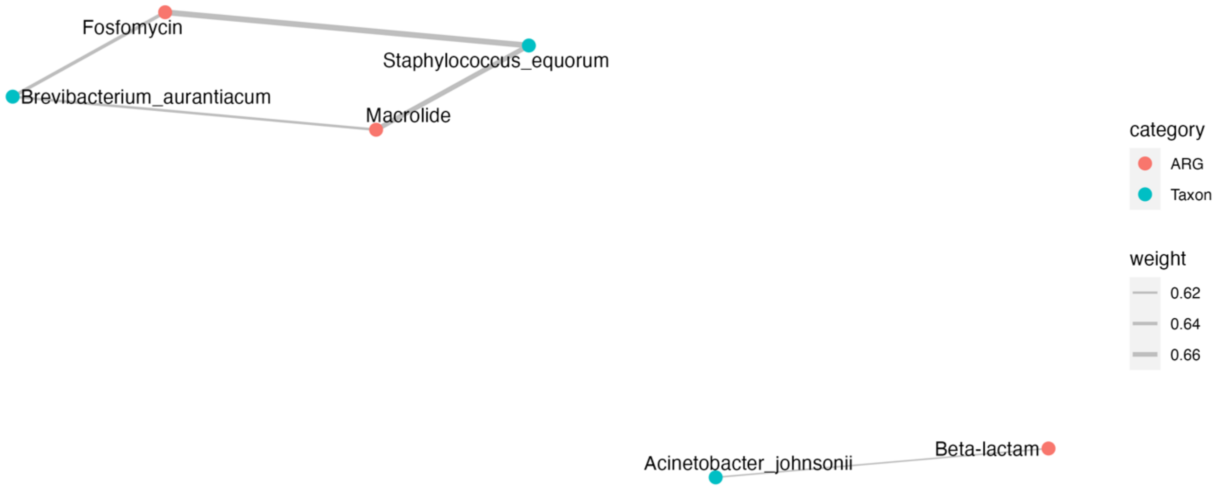

Supplementary Figure 4. Correlation network showing significant Spearman's correlations between the relative abundance of taxa (MetaPlAn profiles) and AMRG families CPM abundance.

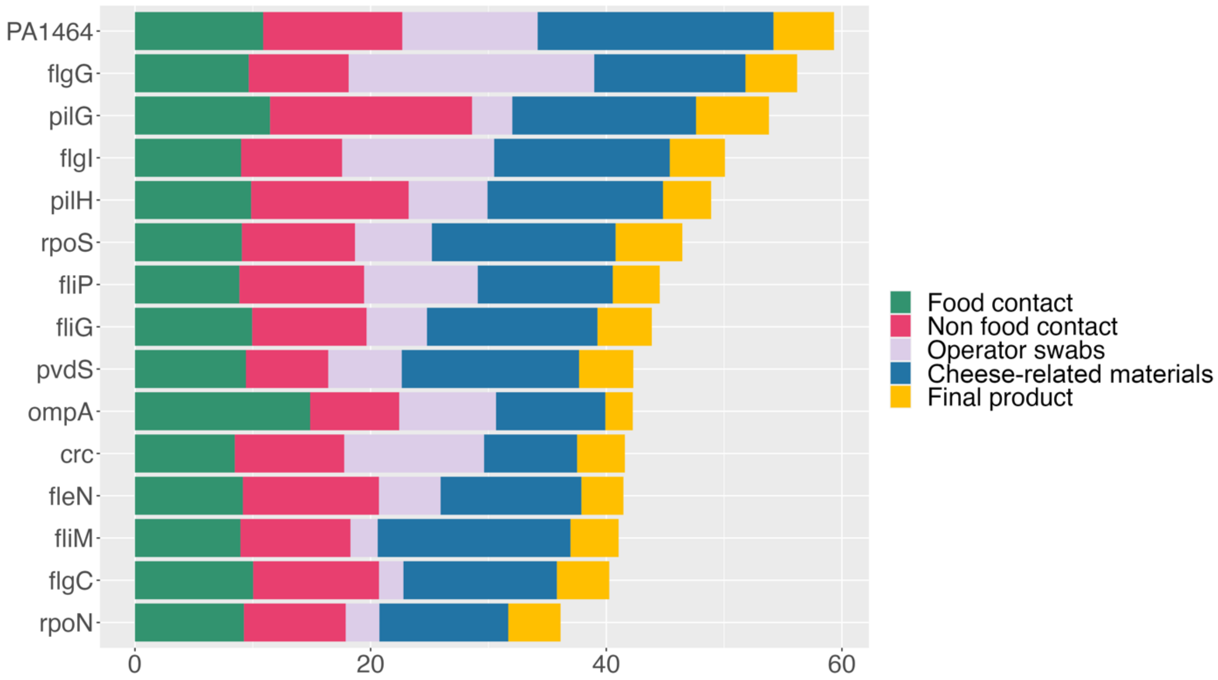

Supplementary Figure 5. Barplot showing the average CPM abundance of the top 15 virulence-associated genes in the groups of samples. The category "cheese-related materials" groups together milk, brine and whey culture.

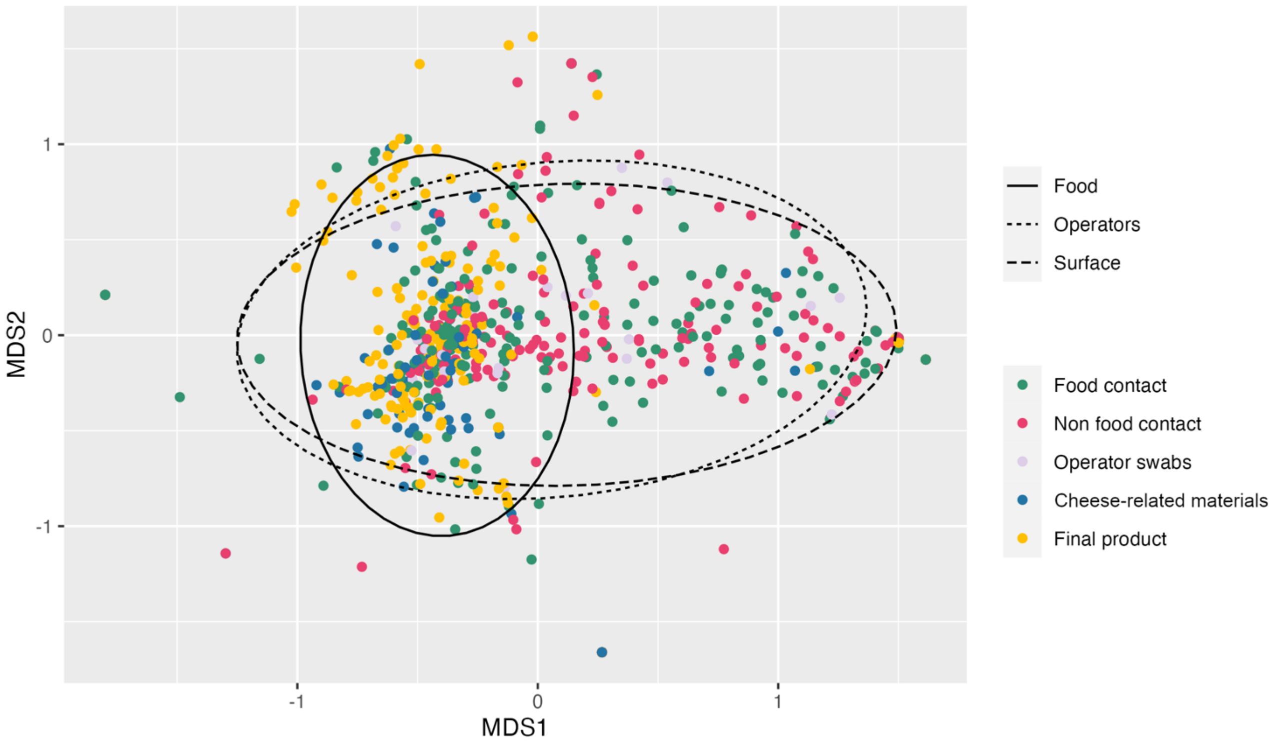

Supplementary Figure 6. Non-metric Multidimensional Scaling (NMDS) based on the Bray-Curtis distance computed on the CPM abundance of virulence families. The category "cheese-related materials" groups together milk, brine and whey culture.

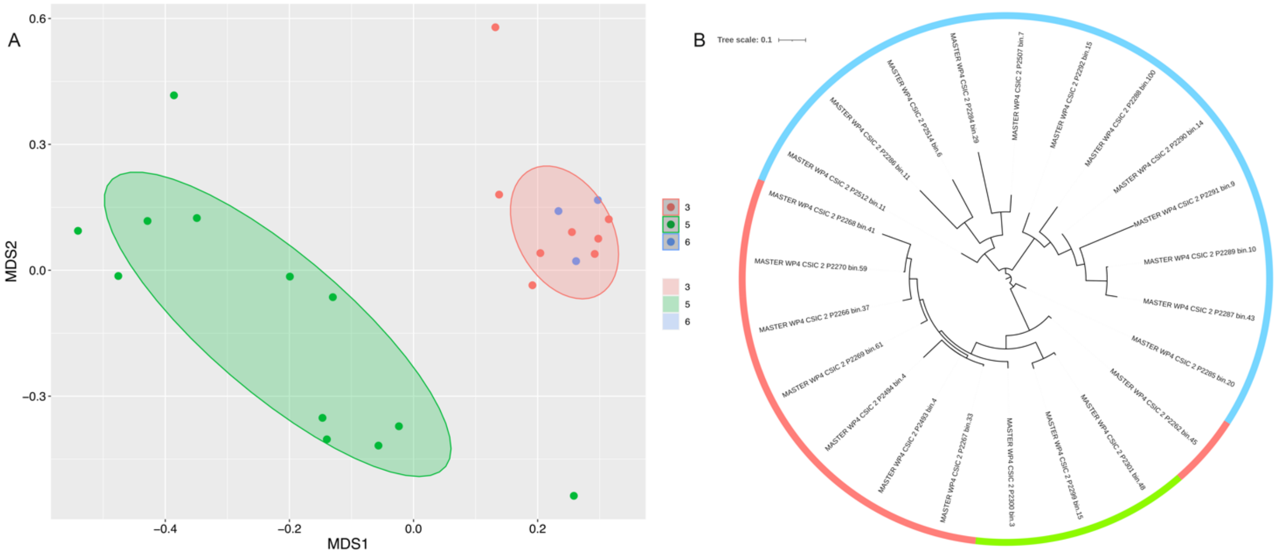

Supplementary Figure 7. A Non-metric Multidimensional Scaling (NMDS) based on ANI distance matrix of MAGs belonging to SGB_12 (*S. thermophilus*) reconstructed from Afuega`l Pitu cheese facilities. Points are colored according to the facility code. **B.** Phylogenetic tree of MAGs belonging to SGB_12 reconstructed from Afuega`l Pitu cheese facilities.

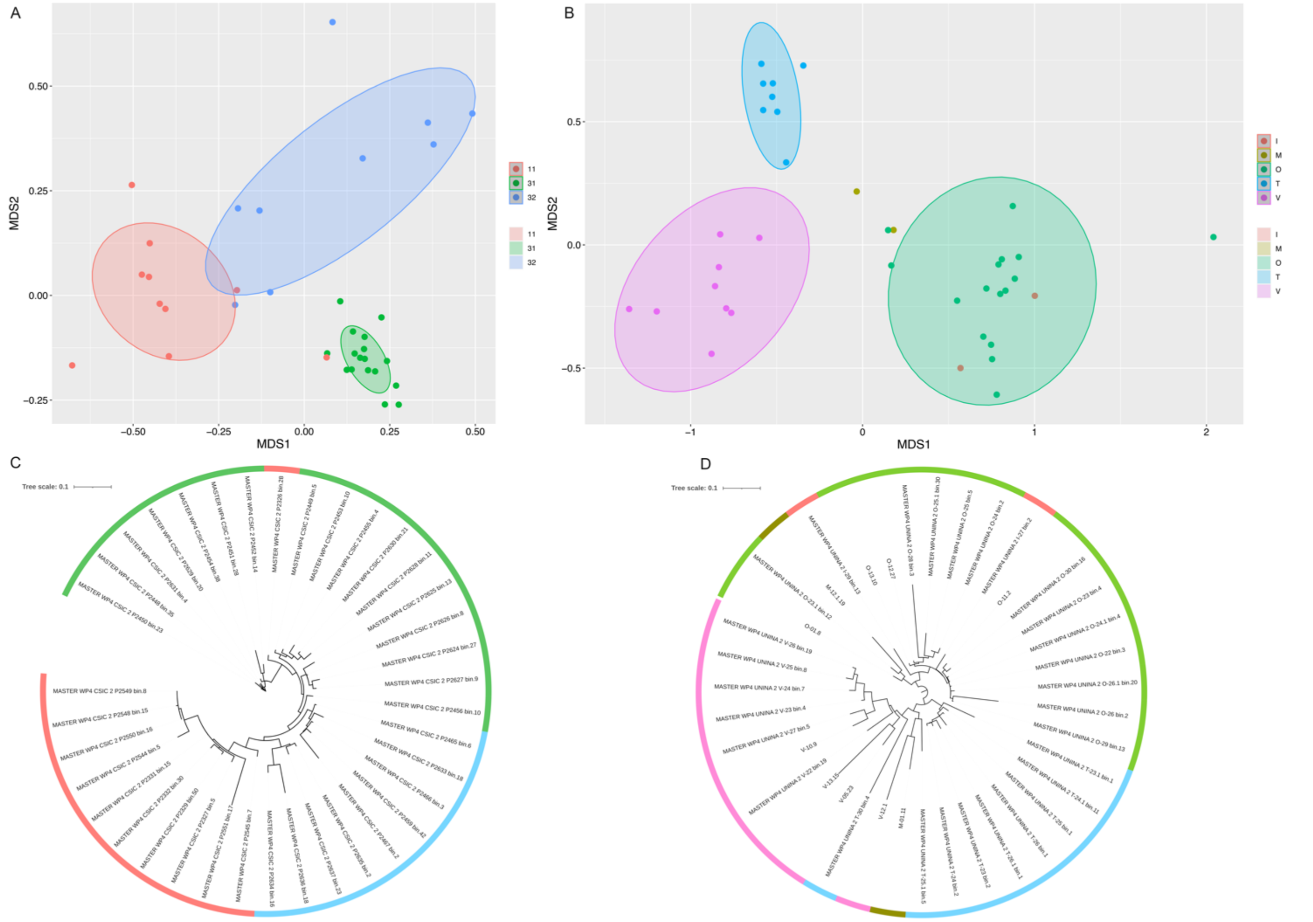

Supplementary Figure 8. A-B. Non-metric Multidimensional Scaling (NMDS) based on ANI distance matrix of MAGs belonging to SGB_73 (*Lc. lactis*) reconstructed from Casín **(A)** and Caciocavallo **(B)** cheese facilities. Points are colored according to the facility code. **C-D.** Phylogenetic trees of MAGs belonging to SGB_73 reconstructed from Casín **(C)** and Caciocavallo **(D)** cheese facilities.

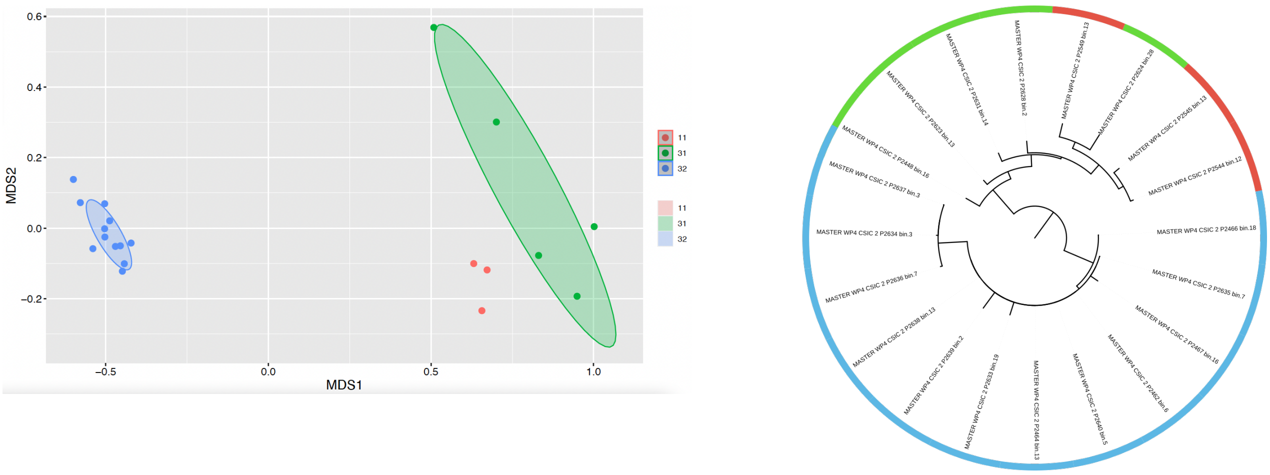

Supplementary Figure 9. A. Non-metric Multidimensional Scaling (NMDS) based on ANI distance matrix of MAGs belonging to SGB_77 (*Leuconostoc mesenteroides*) reconstructed from Casín **(A)** cheese facilities (coded 11, 31 and 32). Points are colored according to the facility code. **B.** Phylogenetic trees of MAGs belonging to SGB_77 reconstructed from Casín cheese facilities.
